# Moving at the frontline

**DOI:** 10.7554/eLife.20516

**Published:** 2016-09-09

**Authors:** Marco Ataíde, Wolfgang Kastenmüller

**Affiliations:** Institute of Experimental Immunology, University of Bonn, Bonn, Germany; Institute of Experimental Immunology, University of Bonn, Bonn, Germanywkastenm@uni-bonn.de

**Keywords:** Innate like lymphocytes, Migration, Lymph node, CD169, SCS Macrophage, IL17, Mouse

## Abstract

The factors that regulate how immune cells called innate-like lymphocytes are localized in the tissue have been identified.

**Related research article** Zhang Y, Roth TL, Gray EE, Chen H, Rodda LB, Liang Y, Ventura P, Villeda S, Crocker PR, Cyster JG. 2016. Migratory and adhesive cues controlling innate-like lymphocyte surveillance of the pathogen-exposed surface of the lymph node. *eLife* **5**:e18156. doi: 10.7554/eLife.18156

To fight off infectious microbes the cells of the immune system – including lymphocytes and monocytes – must migrate to the site of the infection. This concerted response requires an efficient communication process, which usually involves the immune cells releasing cytokines, interferons and other signaling molecules.

The immune system also ensures that some immune cells are already placed at sites at which a pathogen invasion (or re-invasion) may be anticipated (Mueller et al., 2013). These cells include "memory" cells that are associated with the adaptive immune system, such as T and B lymphocytes, and cells that are associated with the innate immune system, including innate lymphoid cells and innate-like T lymphocytes (Fan and Rudensky, 2016). Now, in eLife, Jason Cyster and co-workers at the University of California, San Francisco and the University of Dundee – including Yang Zhang as first author – report the identity of the molecules that coordinate the localization and migration of innate-like T lymphocytes during microbial defense (Zhang et al., 2016).

Innate-like T lymphocytes are a highly diverse group of cells and are typically found at barrier tissues like the gut, the skin or the lung (Godfrey et al., 2015), and in the lymph nodes that drain these barrier tissues. Since pathogens initially spread from infected tissues through the lymph rather than the blood (Trueta and Barnes, 1940), lymph nodes have a critical filter function. In lymph nodes, memory CD8 T cells and innate-like lymphocytes are localized close to the lymphatic sinuses – the sites where invading pathogens may be anticipated (Kastenmüller et al., 2013; Kastenmüller et al., 2012).

Macrophages that line the sinuses and express the surface marker CD169 play a critical role in orchestrating a heterogeneous group of immune cells that defend the lymph node. These macrophages are activated via various pattern recognition receptors (Latz et al., 2013). Activating such receptors leads to the assembly of the so-called inflammasome and to the consecutive cleavage and secretion of two critical cytokines (Schroder and Tschopp, 2010).

The two cytokines – interleukin-18 and interleukin-1β – work with other interleukins to activate different sets of immune cells. Interleukin-18 ultimately activates the immune cells that, in turn, produce an interferon called IFNγ, which acts on macrophages and boosts their capacity to ward off pathogens inside cells. The second cytokine, interleukin-1β, activates a set of innate-like T lymphocytes that respond by secreting another cytokine, interleukin-17. This leads to the recruitment of neutrophils – cells that are particularly critical for the defense against bacteria and fungi.

Zhang et al. analyzed the factors that regulate the migration patterns and localization of the innate-like T lymphocytes. In particular, they studied a group of these cells that have previously been characterized by the Cyster group; these cells reside in the lymph node and express a type of surface receptor called CXCR6 (Gray et al., 2012). Zhang et al. used a number of techniques to study these lymphocytes. The cells were visualized by combining intravital two-photon microscopy in mice with fluorescent confocal microscopy of tissue sections. In addition, antibody labeling tracked the cells that accessed the mouse lymphatic sinuses. Zhang et al. also analyzed the cellular immune response in mice that were exposed to microbial infections.

Strikingly, Zhang et al. found that the innate-like lymphocytes required a surface receptor called CCR6 for proper positioning near the lymphatic sinuses. In the absence of this receptor the innate-like lymphocytes were positioned in deeper areas of the lymph node. In line with this result, Zhang et al. identified lymphatic endothelial cells as the source of CCL20 – the chemokine that is sensed by CCR6 (see Figure 1). During an infection by *S. aureus* bacteria, innate-like T lymphocytes that lack CCR6 produced less interleukin-17 than normal. This occurred because the bulk of these cells were localized too far away to respond to interleukin 1β signals from the macrophages that line the sinuses. Thus to work effectively, innate-like T cells need to be placed close to the CD169-expressing macrophages that line the sinus.

**Figure 1.**
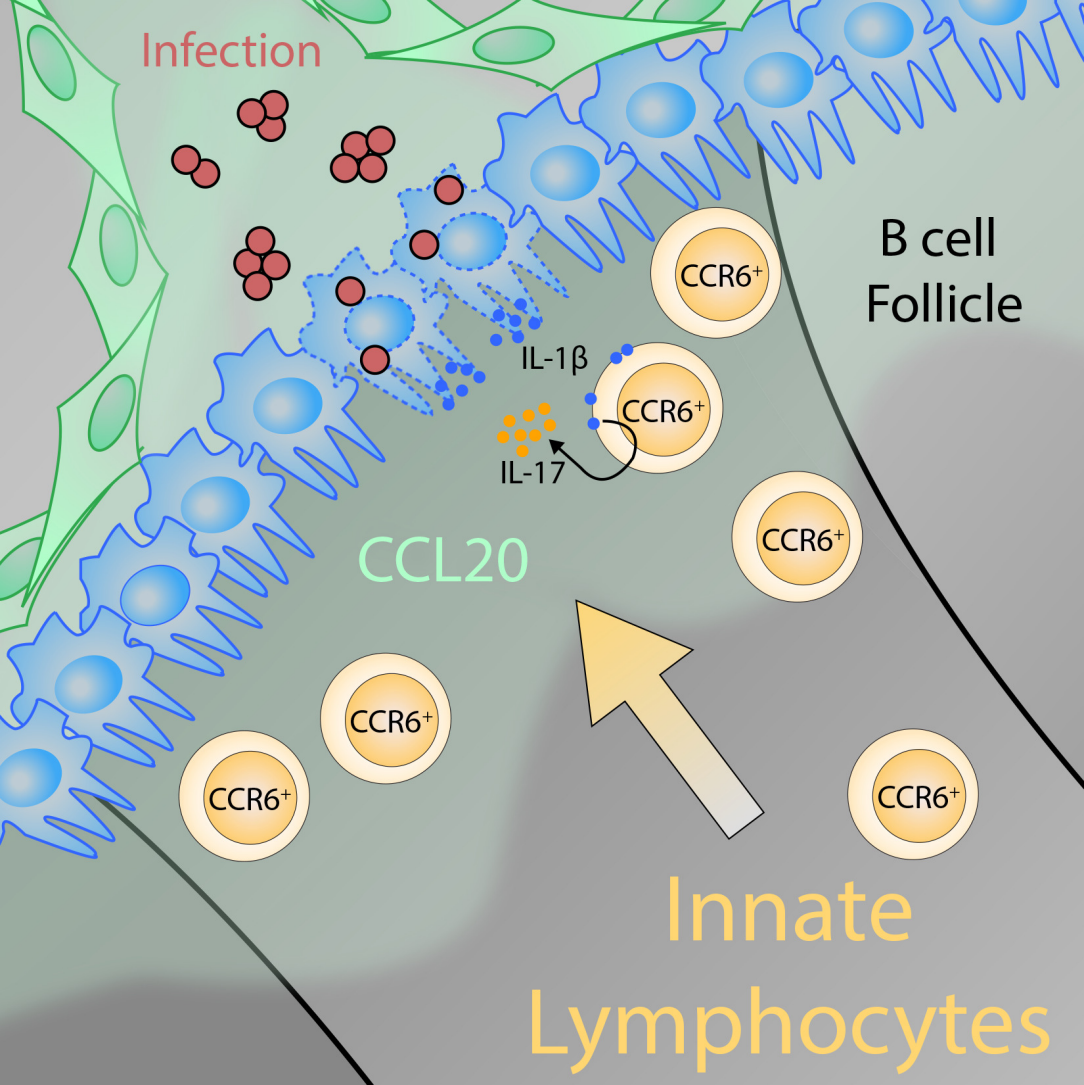
The positioning of innate-like T lymphocytes in the lymph node is crucial for defending against pathogens. Lymphatic epithelial cells (green) release the chemokine CCL20, resulting in a gradient in the lymph node that acts as a cue for innate-like T lymphocytes with CCR6 receptors on their surface (CCR6+; ochre cells) to migrate toward the subcapsular sinus area (the region of the lymph node where lymph enters the tissue). There, the innate-like T lymphocytes can sense interleukin-1β (IL-1β) that the macrophages that line the sinus (blue cells) release locally in response to the activation of the inflammasome. Together with interleukin-23 (not shown), IL-1β activates the CCR6+ innate-like lymphocytes, which in turn produce interleukin-17 (IL-17). IL-17 is a critical factor for microbial defense because it promotes the recruitment of other innate immune cells – neutrophils and monocytes. The graphical illustration was designed and kindly provided by Karl Komander.

A number of other factors (the signaling molecule S1P, and the surface receptors ICAM and CD169) were shown to orchestrate “excursions” of a subset of innate-like T cells (γδ T cells that express Vγ4) into the lymphatic sinuses of the lymph node. The data are compelling, yet the antimicrobial response remained unaltered if these factors were experimentally perturbed. The biological implication of this particular migration pattern therefore requires further investigation.

Overall, Zhang et al. have taken a major step toward a better understanding of the microanatomy of the lymph node and the cellular dynamics of innate-like lymphocytes during homeostasis and after microbial infection. Identifying the factor that regulates the positioning of innate-like T cells has directly demonstrated the functional importance of lymphocyte localization for defending against pathogens.
